# Incorporating Electrolyte Correlation Effects into Variational Models of Electrochemical Interfaces

**DOI:** 10.1021/acs.jpclett.3c03295

**Published:** 2024-02-13

**Authors:** Nils Bruch, Tobias Binninger, Jun Huang, Michael Eikerling

**Affiliations:** † Theory and Computation of Energy Materials (IEK-13), Institute of Energy and Climate Research, Forschungszentrum Jülich GmbH, 52425, Jülich, Germany; ‡ Chair of Theory and Computation of Energy Materials, Faculty of Georesources and Materials Engineering, RWTH Aachen University, 52062, Aachen Germany

## Abstract

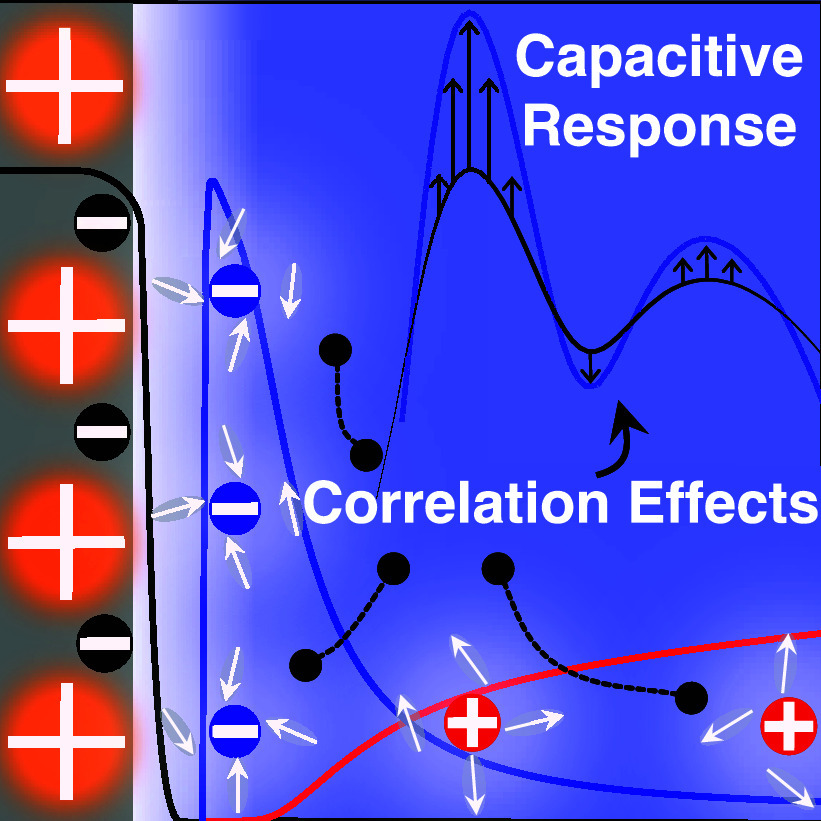

We propose a way for obtaining a classical free energy density functional for electrolytes based on a first-principle many-body partition function. Via a one-loop expansion, we include coulombic correlations beyond the conventional mean-field approximation. To examine electrochemical interfaces, we integrate the electrolyte free energy functional into a hybrid quantum-classical model. This scheme self-consistently couples electronic, ionic, and solvent degrees of freedom and incorporates electrolyte correlation effects. The derived free energy functional causes a correlation-induced enhancement in interfacial counterion density and leads to an overall increase in capacitance. This effect is partially compensated by a reduction of the dielectric permittivity of interfacial water. At larger surface charge densities, ion crowding at the interface stifles these correlation effects. While scientifically intriguing already at planar interfaces, we anticipate these correlation effects to play an essential role for electrolytes in nanoconfinement.

Electrified interfaces in contact with electrolytes are ubiquitous in soft matter physics, biology, and electrochemistry.^1−5^ In the latter realm, the microscopic region of the electric double layer (EDL) controls the capacitive response of the interface as well as the kinetics of quintessential electrocatalytic reactions.^6,7^ The EDL thereby controls the performance of electrochemical energy devices.^8,9^ Understanding and predicting the all-important local reaction environment at the interface is of primary interest in this field. To achieve this, we must handle the interplay of metal electronic structure, adsorbates, solvent molecules, and ionic species, with all of these effects treated self-consistently as a function of electrode potential.^10−14^ Efforts in theory and simulation strive to find a solution to this problem.^15−17^

Using quantum-mechanical density functional theory (DFT) for both metal and electrolyte is an intriguing option, but remains impractical for most systems of interest even with today’s massive computational resources. As a way out of this dilemma, the majority of methods utilize some kind of hybridization scheme that describes electrode and electrolyte regions at distinct levels of theory.^18^ While electronic effects in the metal must be treated quantum mechanically at the level of DFT, treatments of electrolyte effects vary from force field-based molecular dynamics methods (MD)^19−21^ to approaches based on continuous density distributions for solvent and ions.^7,22−24^

Two sophisticated hybrid schemes using implicit solvation have emerged. One of these, the DFT/ESM-RISM approach, combines Kohn–Sham DFT (KSDFT) with Ornstein–Zernike (OZ) integral equation theories in the reduced interaction-site model (RISM) framework and the electrostatic screening medium (ESM) technique.^25−29^ The second hybrid scheme employs KSDFT for the metal and classical DFT for the electrolyte side which is minimized using a variational principle.^30,31^ The complexity of electrolyte density functionals ranges from polarizable continuum models^32−34^ to molecular DFT (MDFT).^35,36^ Such methods to simulate electrochemical interfaces are still computationally expensive, especially when performed at fixed electron chemical potential, i.e., using the grand canonical ensemble, which is the appropriate ensemble to mimic experimental conditions.^18,37^ A more pragmatic ansatz termed density-potential functional theory (DPFT), treats the metal side at the level of orbital free DFT and has achieved qualitative agreement with experimental capacitance data over a wide range of electrochemical potentials.^17,38−40^

In these approaches, the free energy density functional for the electrolyte embodies the description of the EDL. In the case of a dilute aqueous electrolyte at a weakly charged metal surface, the well-known mean-field free energy leads to the Poisson–Boltzmann model, which represents a sound approach to calculating electric field and ion density distributions. Refined functionals incorporate steric effects due to hardcore repulsion, as is the case for the modified PB model (MPB).^41,42^ The approach developed by Bazant et al. explains overscreening at small surface charge density. Combined with the MPB equation, it describes ionic overcrowding at high surface charge density.^43,44^

In mean-field models, it is assumed that an electrolyte particle only interacts with an averaged electric field created by all other charge carriers. Coulombic interactions among ions beyond mean-field, such as ion-pairing or screening, are referred to as coulombic correlation effects. They constitute a notoriously challenging class of phenomena to be handled by density functionals. Coulombic correlation effects have been identified as the cause for peculiar findings in charged systems. A prime example of coulombic correlation effects is the smaller than one activity coefficient of bulk electrolytes as described within Debye–Hückel (DH) theory.^45^ Another striking observation is the attraction of two similarly charged plates in contact with monovalent counterions in between.^46,47^

Electrolyte correlation effects are commonly treated by the Ornstein–Zernike integral equations,^48^ typically solved in hypernetted chain approximation,^49−55^ as implemented in RISM.^56−58^ Ramirez et al. presented an ansatz to construct a free energy functional for MDFT by inverting the Ornstein–Zernike equation where the two-body distribution functions are computed from MD simulations.^35,36^

An alternative approach to treating correlation effects involves an initially exact statistical mechanics-based formulation.^59^ Netz and Orland used a functional integral formalism and, based thereon, unveiled correlation effects with the help of a loop expansion for a one-component plasma near a charged surface. This elegant approach allows including fluctuation effects consistently.^60−62^ Following these works, the interest in correlation effects spiked, and their impact on relevant bulk and interface properties was assessed.^63−71^ Other studies harnessing this framework aimed at elucidating solvent structures.^71−75^

Merging the functional integral formalism with efficient variational approaches for the EDL appears to be a very promising, albeit mathematically intricate, endeavor. To the best of our knowledge, this feat has not been accomplished to date.

In this letter, we derive a variational method from the many-body partition function to incorporate correlation effects into a classical density functional theory for the electrolyte. In this way, limitations of the traditional mean-field approaches can be overcome. The derived free energy functional is then embedded into the established DPFT framework, for simulating electrolyte correlations at electrochemical interfaces under constant electrode potential. We find that the mean-field interaction between electrolyte and electrostatic potential can be generalized to include coulombic correlation effects by introducing a single scaling function for each particle species. The scaling functions generalize DH theory correlations to nonuniform systems. Like DH theory, the scaling functions are greater than one at constant chemical potential, resulting in an increased counterion density. We then study the capacitive response of a planar metal–electrolyte interface in the presence of electrolyte correlations. Due to the increase of the counterion density, we find a trend that the differential capacitance is significantly increased in comparison to the mean-field prediction. It is only at larger surface charge densities, where steric effects become important, that the one-loop corrections disappear.

The grand potential that encapsulates interactions and correlations among components of the metal–electrolyte interface can be written in general form as,
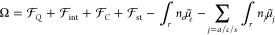
1where ∫_*r*_ is short-hand notation for ∫ d^3^*r*, where *r* is a three-dimensional vector. The variable *n*_*j*_ denotes the local density, and μ̃_*j*_ represents the applied electrochemical potential for anions (a), cations (c), or solvent (s). Similarly, *n*_*e*_ and μ_*e*_ correspond to the electron density and applied electrochemical potential, respectively. The grand potential is the Legendre transform of the interface free energy consisting of a quantum mechanical part for the electronic subsystem in the metal, , a classical potential functional, , to describe electrostatic interactions between electrolyte particles, an interaction free energy, , which describes metal–electrolyte interactions, and a free energy functional, , to account for steric effects.^17,38−40^

We present now an approach to derive the Coulomb free energy  including correlation effects. Following the derivation of Podgornik, we express the grand canonical partition function  for the electrolyte as an integral over all possible configurations of the electrolyte.^59^ In most cases, this integral is too complex to be evaluated exactly. However, Podgornik showed that, using a Hubbard–Stratonovic transformation, the partition function can be mapped exactly to a functional integral,
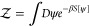
2where β = 1/(*k*_B_*T*) is the inverse temperature, and *k*_B_ the Boltzmann constant. Here, integration is performed over a single auxiliary potential field ψ(*r*) that spans the domain of interest, with each configuration weighted by the exponential of an action functional *S*[ψ] that entails interactions between electrolyte particles.

The electrolyte considered in the present work consists of anions and cations, modeled as point-like charge carriers with density *n*_*i*_ and charge *q*_*i*_. The solvent is a dipolar fluid with density *n*_*s*_ and dipole moment of fixed strength *p⃗*. The only interactions considered are Coulomb interactions. For a given electrolyte composition, we have derived the nonlinear action to be of the form,
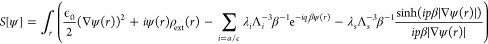
3where Λ_*j*_ are thermal wavelengths.^76^ In a similar form, this action appeared in the dipolar-Poisson–Boltzmann model.^77^ The electrolyte system is coupled to a reservoir to fix the chemical potentials of electrolyte species, μ_*j*_, related to fugacities, λ_*j*_ = exp(*βμ*_*j*_). The first two terms on the right-hand side of eq 3 encompass the kinetic energy of the auxiliary field and its interaction with an external charge density ρ_ext_(*r*), which is used within this approach to model a mean-field coupling of the electrostatic potential to the electron density of the metal. The second line contains the interaction of charge carriers of the electrolyte, with fugacities λ_*i*_, with the auxiliary field. The third line incorporates interactions of the dipolar density, with fugacity λ_*s*_, with the gradient of the auxiliary field.

The partition function of eq 2 with the action functional of eq 3, and thus the grand canonical free energy , cannot be evaluated analytically. We therefore need to rely on approximations. The loop-wise expansion is a series expansion around the saddle-point (mean-field) of the action, as employed by Netz and Orland.^60^ It maps the functional integral of eq 2 onto a variational functional for the electric potential. This approach captures fluctuations of the electrostatic potential relative to the mean-field solution. It allows describing correlation effects systematically with an accuracy that can be increased order by order. The one-loop expansion is the lowest nontrivial order beyond mean-field (zeroth order) that captures quadratic fluctuations. This approximation yields a free energy variational functional of the form,
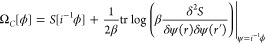
4where ϕ(*r*) is a field. If it fulfills the variational equation
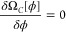
5ϕ(*r*) is equal to the electrostatic potential. While the first term on the right-hand side of eq 4 represents the mean-field approximation, the second term encodes correlation effects to one-loop order. The derivation of the variational functional eq 4 is given in the Supporting Information (SI). It has been shown by Netz and Orland that the one-loop expansion is valid when the Gouy–Chapman length exceeds the Bjerrum length, which is the case for low-valent electrolytes and small surface charge density.^60,64^ As a result, the one-loop expansion is accurate around the potential of zero charge (pzc), which is the focus of interest in this letter.

To couple the electrolyte theory to a quantum mechanical density functional of metal electrons as in eq 1, we need an expression for the electrolyte free energy that is a functional of particle densities and not chemical potentials (fugacities λ_*j*_). The requisite Legendre transform to the canonical ensemble is defined by,
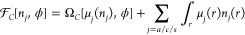
6where chemical potentials and particle densities are related via
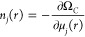
7Note that the chemical potentials in eq 7 are spatially dependent. With this transformation, we shift from an equilibrium free energy function of chemical potentials to a variational functional for particle densities. The details of this transformation are presented in the SI. For the action in eq 3, using the result for the grand canonical free energy in eq 4 and inverting the relation eq 7, we find
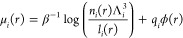
8
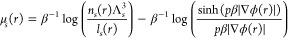
9The first expressions on the right-hand side of eqs 8 and 9 represent chemical contributions. Densities in these contributions are scaled by dimensionless correlation parameters, defined as,
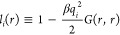
10
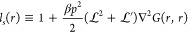
11where, omitting the argument from now on,  and  are the Langevin function and its first derivative, respectively, and *u* = *pβ*|∇ϕ|. These parameters encode the corrections due to correlation effects to one-loop order. The second terms in eqs 8 and 9 account for the usual mean-field coupling of the electrolyte to the electric potential.^38^ Introducing *l*_*i*_ and *l*_*s*_ allows interpreting the impact of correlations using a single parameter for each particle species. For details regarding the derivation of the chemical potentials and the correlation parameters, we would like to refer the reader to the SI.

The Green’s function, *G*, is defined as the (operator) inverse of the second variational derivative of the action functional, cf. eq 4. It solves the following differential equation,
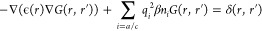
12with . This differential operator describes the correlations between particles. It accounts for screening in the presence of dipoles, as can be seen by setting the solvent density *n*_*s*_ = 0, for which the Green’s function *G*(*r*, *r′*) that solves eq 12 reduces to a simple screened Coulomb potential. This differential equation is a generalization to the equation derived by Netz and Orland. In addition to that prior variant, it accounts for the presence of salt and point-dipoles.^60^ To obtain an analytical approximation for *G*, we neglect the spatial dependencies in eq 12 and introduce a small distance cutoff Λ_B_ to render the correlation function finite at equal argument, as required in eq 10. The divergence is due to the breakdown of the continuum field theory below a certain length scale. Physically, the cutoff Λ_B_ is the length-scale, above which coulombic correlation effects are included.^78^ Thereby, we account for local correlation effects between solution particles as in bulk systems but not due to spatial inhomogeneities in densities or dielectric constant.^62,64^ Details of correlation parameters for the given correlation function can be found in the SI.

Coming back to the Legendre transform in eq 6, we insert the results for the chemical potentials from eqs 8 and 9 to obtain an expression for the free energy of the electrolyte system,
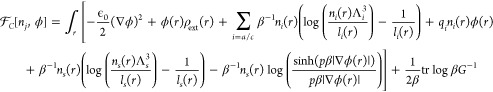
13This expression is the key novel result of this letter. It generalizes the usual mean-field coupling between the electrostatic potential and the electrolyte densities, by including correlation effects in the form of the scaling functions *l*_*i*_(*r*) and *l*_*s*_(*r*) defined in eqs 10 and 11, respectively. Setting *G* to zero, the free energy reduces to the mean-field result as required.^17,40^

We now combine the free energy functional of eq 13 with the DPFT interface functional of eq 1, which allows us to investigate coulombic correlation effects at metal–electrolyte interfaces self-consistently. The DPFT framework offers the key advantage of yielding electron density and potential distributions of the interface under constant potential conditions, from which all other properties of the double layer can be derived. Electrons are described within the DPFT framework by an orbital free density functional, , comprising the kinetic energy *T*_*e*_ from Thomas–Fermi theory and the exchange correlation parts *U*_*ex*_ and *U*_*C*_ from the Perdew–Burke–Ernzerhof functional.^79,80^ For the atomic cores, a jellium model of constant positive charge density is used. This representation for the quantum mechanical system is useful since it enables coupling degrees of freedom of metal electrons self-consistently with degrees of freedom of electrolyte species, while being, at the same time, computationally efficient. It should be noted, however, that the present formalism does not account for correlation between metal electrons and electrolyte species.^81^

The repulsive interaction between metal and solution phase, , using Lennard-Jones potentials, prevents solution species from entering the metal. Additionally, the model accounts for hard-core interactions between solution particles at the level of Bikerman theory for equal size particles on a lattice with site density *n*_max_,^41,42^ which results in the steric free energy term  Details of the functionals used can be found in the SI. Finally, we arrive at the full grand potential in eq 1.

Minimization of the grand potential, using the variational principle, results in a set of five coupled equations,
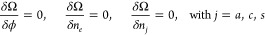
14Since the respective equations for the densities of electrolyte species do not contain any gradient terms, one obtains a system of three linear equations that can be solved analytically, leading to the following density expressions,
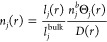
15where Θ_*i*_(*r*) = exp(−*q*_*i*_*βϕ*) and  are Boltzmann factors for ions and solvent, respectively, and *D*(*r*) = (1 – ∑_*j*_*n*_*j*_^*b*^) + ∑_*j*_*l*_*j*_(*r*) / *l*_*j*_^*b*^ · *n*_*j*_^*b*^Θ_*j*_(*r*), with *n*_*j*_^*b*^ being the bulk density of the electrolyte, takes into account the finite size of particles.

Eq 15 constitutes an intuitive result originating in correlation effects. In the bulk, where *l*_*j*_(*r*) = *l*_*j*_^bulk^ and the Boltzmann factors are one, we simply obtain the bulk density. The difference to the mean-field model from Huang et al. is the positive prefactor *l*_*j*_/*l*_*j*_^bulk^ that renormalizes the density at the interface and is a direct consequence of the second term in eq 4. This shows that correlation effects for one particular species *j* are exclusively encoded in *l*_*j*_, which are determined for ions by the Green’s function *G* and for the solvent by ∇^2^*G*, cf. eqs 10 and 11.

To obtain the electron density and the electric potential, we must solve a system of two coupled partial differential equations. For the electronic equation, we refer to earlier works.^38^ The equation for the electric potential can be written in the form of a modified Poisson–Boltzmann equation,
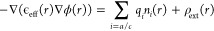
16with ρ_ext_(*r*) = *e*(*n*_cc_(*r*) – *n*_*e*_(*r*)) the total charge density of the metal, composed of the electron density *n*_*e*_ and the constant positive background *n*_cc_, where *e* denotes the elementary charge. The factor ϵ_eff_(*r*) corresponds to the effective dielectric permittivity,
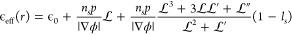
17This form of the permittivity shows a behavior similar to the phenomenological model of Grahame,^82^ where the dielectric constant, for vanishing electric field, assumes the value for bulk water and approaches the vacuum permittivity for very high electric fields. The first two terms on the right-hand side of eq 17 represent the known mean-field result, where the dielectric permittivity couples linearly to the dipole density and the electric field through the Langevin function.^77^ The third term in eq 17 embodies correlation effects and exhibits a decrease in permittivity with increasing salt concentration, cf. the form of *l*_*s*_ in eq S.31, consistent with prior one-loop calculations and experimental studies.^63,83−85^

We now consider an aqueous 1:1 electrolyte in the form of KPF_6_ solvated in water in contact with Ag(111) electrode. The values of parameters are listed in the SI. The electrode potential in this model is the applied electron chemical potential *E* = *e*^–1^μ̃_*e*_. A typical field distribution for *E* = 0.6 V vs *E*_pzc_ is shown in Figure 1, where *E*_pzc_ is the potential of zero charge (pzc) of the electrode. Due to a positive electrode potential relative to the pzc, a positive surface charge density is present on the metal. This results in the attraction of anions and the repulsion of cations from the metal surface, as indicated by an elevated anion density. To examine the impact of the correlation function on field distributions, we performed calculations for the same chemical potentials, but with a very large cutoff, implying the absence of correlations. This approach allows us to quantify corrections to the fields due to correlation effects, e.g., changes in potential ϕ^1L^ – ϕ^MF^, where the superscript indicates whether correlations are switches on (1L) or off (MF). The simulation results for a 100 mM electrolyte for electrode potentials from *E* = +0.6 to −0.4 V vs *E*_pzc_, are shown in Figure 2. The changes in the electric potential distribution resulting from the one-loop expansion are depicted in Figure 2a. This figure reveals a reduction in local potential for a positive electrode potential, suggesting a diminished anion density in accordance with the Boltzmann factor for anions, cf. eq 15. However, we observe that the counterion density, shown in Figure 2c, is elevated relative to the mean-field result near the interface and diminished further away due an overestimation of interionic Coulomb repulsion within the mean-field reference.^60,62^ This deviation is due to the scaling function *l*_*a*_/*l*_*a*_^bulk^, that renormalizes the counterion density, for a positive electrode potential, cf. eq 15. We find *l*_*a*_/*l*_*a*_^bulk^ is significantly larger than one (not shown), overcompensating for the reduced local electrostatic potential and leading to a net concentration increase. The increase in counterion density is expected because coulombic electrolyte bulk correlations of DH type are used at the interface, as shown in eqs S.30 and S.31. According to DH theory, the density is higher at a constant chemical potential, due to a reduced activity coefficient caused by screening.^45^

**Figure 1:**
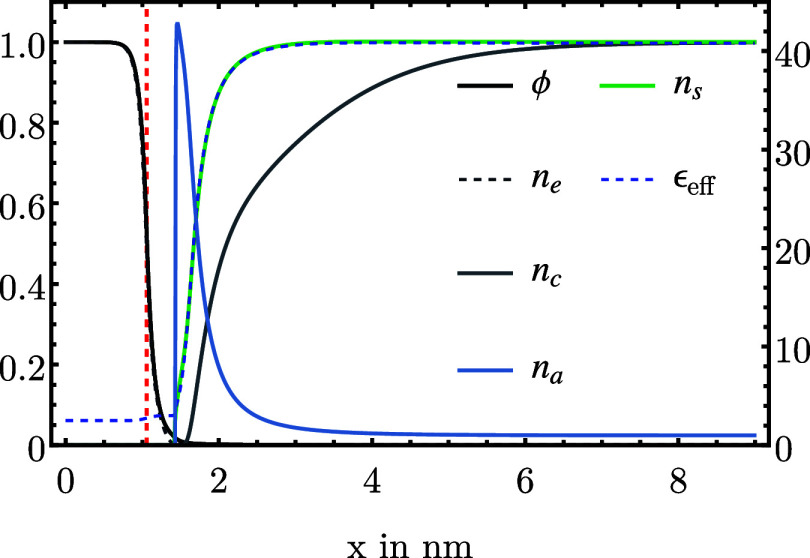
Variation of electron, ion, and solvent densities, electric potential, and dielectric permittivity across the metal–electrolyte interface at fixed electrochemical potential μ̃_*e*_ = −3 eV (*E* = 0.6 V vs *E*_pzc_) and bulk electrolyte concentration *n*_ion_ = 100 mM, obtained by self-consistent solution of eq 14. The red dashed curve indicates the metal boundary. Distributions are normalized to their respective bulk value (metal or solution). The tick marks for the anion concentration (solid blue line) are on the right side, whereas the tick marks for all other variables are on the left side.

**Figure 2:**
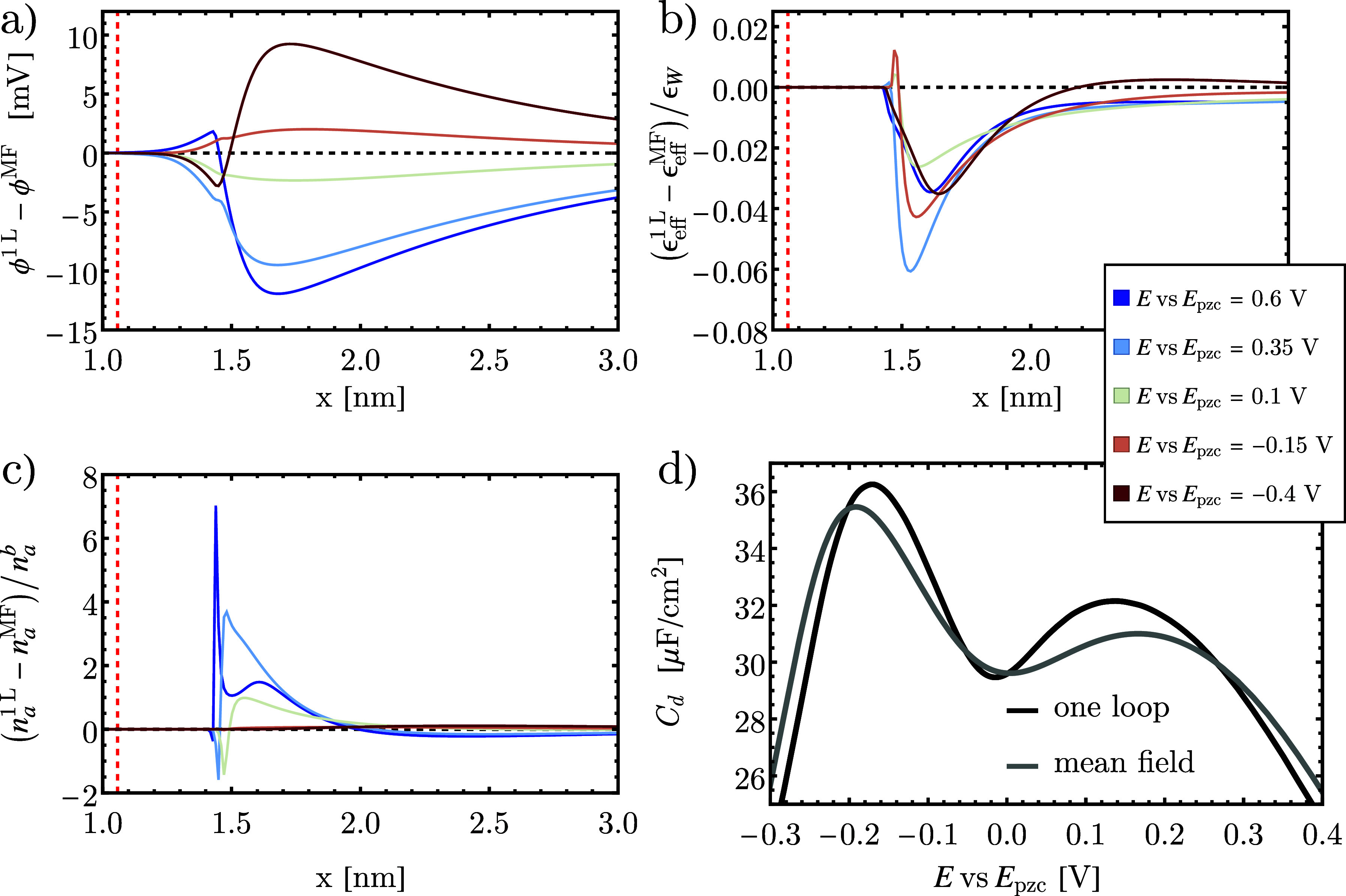
Modeling results at different electrode potentials from *E* = +0.6 to −0.4 V vs *E*_pzc_: (a) the electric potential corrections, (b) permittivity corrections ϵ_eff_^1L^ – ϵ_eff_^MF^ normalized to bulk water permittivity, and (c) anion density corrections normalized to ionic bulk density. Comparing the differential capacitance (d) of the double layer in the mean-field and the one-loop case reveals significant enhancements in the characteristic features of the capacitance curve due to electrolyte correlation effects.

In Figure 2b, a decrease in overall permittivity is observed. This reduction can be attributed to the elevated counterion density, leading to a displacement of solvent from the interface (steric effect), which is more pronounced than the dielectric reduction due to coulombic correlation effects, i.e., the third term in eq 17. The magnitude of the reduction against the mean-field solution varies slightly, with a smaller correction near the pzc and a larger correction at larger surface charge densities. Interestingly, corrections in Figure 2b are the largest at intermediate chemical potentials, i.e., for *E* ≈ – 0.15 V vs *E*_pzc_ and *E* ≈ 0.35 V vs *E*_pzc_, indicating that correlation effects play an important role at partially charged interfaces.

Our results demonstrate that correlation effects exert a significant impact on the shape of the differential capacitance curve. The reduction in permittivity alone would result in a reduced differential capacitance of the EDL, which is observed at the pzc, where the excess surface charge density is zero, cf. Figure 2d. At low surface charge density, however, the elevated counterion density overcompensates the lowered interface permittivity and results in an elevated differential capacitance as a result of correlation effects. At larger electrode potential, the saturated volume close to the interface prevents further accumulation, which limits ion correlation effects. Thus, at very large surface charge densities, the difference between mean-field and one-loop vanishes. Previous studies in the DPFT framework have demonstrated a good agreement between the mean-field model and experimental data for the differential capacitance,^40^ albeit with quantitative discrepancies in features of the capacitance curve such as peak-height and peak-to-peak distance. Our results with coulombic correlation effects lead to larger capacitance values and a more pronounced double-peak structure with higher peaks and shorter peak-to-peak distance compared to the mean-field prediction. These trends are in agreement with experimental data.^40^

In summary, we have presented a novel approach to incorporate electrolyte correlation effects from first-principles into a variational functional formalism for the description of metal–electrolyte interfaces. The derived variational functional captures coulombic correlation between electrolyte particles solely encoded in one parameter for each particle species, which generalizes the mean-field interaction between electrostatic potential and electrolyte densities. Application within the DPFT framework suggests a significant increase in interfacial capacitance due to a correlation-induced increase of counterion densities, which is partially compensated by a reduction of the local permittivity at the interface. At higher surface charge densities, correlation effects are suppressed due to volume exclusion.

While coulombic correlation effects revealed are significant and of general interest, we expect these effects to play a more significant role in nanoconfined geometries.^46,47,59,86−89^ The ramifications of electrolyte correlation effects on ion and solvent phenomena in nanoporous media with charged walls constitute the focus of our forthcoming work—with the methodical basis for any such exploration laid in this letter.

## Supporting Information Available

Additional details regarding the transformation to the variational principle, derivations of results, and model specifications (PDF)
