# Supplementary material for: Incorporating Electrolyte Correlation Effects into Variational Models of Electrochemical Interfaces
Source: J Phys Chem Lett. 2024 Feb 13;15(7):2015–22. doi: 10.1021/acs.jpclett.3c03295 (PMC10895655; doi:10.1021/acs.jpclett.3c03295)
Supplement: Supplementary file 1 — jz3c03295_si_001.pdf [file jz3c03295_si_001.pdf]

Supporting Information: Incorporating Electrolyte Correlation Effects into Variational Models of Electrochemical Interfaces

Nils Bruch,[†,‡] Tobias Binninger,[†] Jun Huang,[†,‡] and Michael Eikerling[*,†,‡]

†*Theory and Computation of Energy Materials (IEK-13), Institute of Energy and Climate Research, Forschungszentrum Jülich GmbH, 52425 Jülich, Germany*

‡*Chair of Theory and Computation of Energy Materials, Faculty of Georesources and Materials Engineering, RWTH Aachen University, 52062 Aachen, Germany*

E-mail: m.eikerling@fz-juelich.de

Contents

1 The derivation of the variational principle **S-2**

2 Derivation of the chemical potentials **S-6**

3 Derivation of the second variational derivative **S-7**

4 Details on the correlation parameters **S-8**

5 Details of free energy functionals **S-9**

6 Model parameters **S-11**

References **S-12**

1 The derivation of the variational principle

The goal of computing the grand canonical free energy and therefore the partition sum from Eq. (2) is, as mentioned in the main text, in general impossible to do exactly. It was, however, shown by Netz and Orland, that within the functional integral formalism, we can use the loop expansion method to transform the functional integral Eq. (2) into a variational functional for the electrostatic potential.

It is possible to show that the electrostatic potential is the functional average over the auxillary field,

$$\phi(r) \equiv i\langle\psi(r)\rangle, \tag{S.1}$$

because ϕ solves the Poisson equation for the ensemble averaged particle density as shown in Eq. (58) of the article by Netz and Orland.[S1] The average,

$$\langle ...\rangle = \frac{1}{\mathcal{Z}}\int D\psi (...) e^{-\beta S[\psi]}, \tag{S.2}$$

is a functional average with the action, Eq. (3), as probability distribution. The electric potential within functional integration can be calculated by adding a fictitious source term to the action in Eq. (2),

$$\mathcal{Z}[\rho_{\text{aux}}] = \int D\psi e^{-\beta S[\psi] + i\int_r \rho_{\text{aux}}(r)\psi(r)}, \tag{S.3}$$

that couples an auxiliary charge density to the auxiliary field. Accordingly, the grand canonical free energy becomes $\Omega_C[\rho_{\text{aux}}] = -\beta^{-1}\log\mathcal{Z}[\rho_{\text{aux}}]$, which is only equal to the old one for $\rho_{\text{aux}} = 0$. Taking the derivative with respect to ρ_{aux} allows one to calculate the electric

potential through

$$\phi(r) \equiv i\langle\psi(r)\rangle = \frac{1}{\beta}\frac{1}{\mathcal{Z}}\left.\frac{\partial \mathcal{Z}}{\partial \rho_{\rm aux}(r)}\right|_{\rho_{\rm aux}=0}. \tag{S.4}$$

The first equal sign is the reason why the electrostatic potential can be called a mean-field. The Legendre transformation, that changes the active variable in the calculation of the partition function $\mathcal{Z}[\rho_{\rm aux}]$ from $\rho_{\rm aux}$ to ϕ, gives the so called generating functional, effective action or thermodynamic potential,

$$\Omega_C[\phi] = \Omega_C[\rho_{\rm aux}] + \int_r \phi(r)\rho_{\rm aux}(r), \tag{S.5}$$

which by construction, exactly satisfies the variational equation,

$$\frac{\delta\Omega_C[\phi]}{\delta\phi} = 0, \tag{S.6}$$

which states that the proper electrostatic field configuration makes the functional $\Omega_C[\phi]$ stationary. If one now inserts the partition function from Eq. (S.3) into the definition of the generating functional Eq. (S.5) and exponentiates both sides, one arrives after a shift of integration at the important relation,

$$e^{-\beta\Omega_C[\mu_j,\phi]} = \int D\delta\psi \; \exp\Big(-\beta S[i^{-1}\phi + \delta\psi] + i\beta\int_r \delta\psi(r)\rho_{\rm aux}(r)\Big). \tag{S.7}$$

Note that until now, the formalism does not involve approximations, rendering the theory exact. However, the functional integral is still impossible to solve.

In the one-loop expansion, the action is expanded around the mean-field ϕ in fluctuations

$\delta\psi$ until second order,

$$
\begin{aligned}
e^{-\beta\Omega_C[\phi]} = & \int D\delta\psi \exp\Bigg(- \beta S[i^{-1}\phi] \\
& - \beta \int_r [\frac{\delta S}{\delta\psi(r)} - i\, \rho_{\text{aux}}(r)]\Bigg|_{\psi=i^{-1}\phi} \delta\psi(r) \\
& - \frac{\beta}{2} \int_{r,r'} \delta\psi(r)\, \frac{\delta^2 S}{\delta\psi(r)\delta\psi(r')}\Bigg|_{\psi=i^{-1}\phi} \delta\psi(r')\Bigg) \\
= & \exp\left(-\beta S[i^{-1}\phi] - \frac{1}{2}\text{tr}\log\beta\, \frac{\delta^2 S}{\delta\psi(r)\delta\psi(r')}\Bigg|_{\psi=i^{-1}\phi}\right),
\end{aligned}
\tag{S.8}
$$

where it was used that the linear variation vanishes because the mean-field ϕ is up to one-loop order also the saddle-point of the action. In the last line, a general Gaussian functional integral was performed, neglecting the constant factor. By re-exponentiating, one arrives at the last line. A comparison of the left and right hand side of Eq. (S.8) yields the result for the generating functional in one-loop approximation in Eq. (4).

Eqs. (4) and (5) constitute the transition from a thermodynamic equilibrium function to a free energy functional of a varying field, ϕ, where only the physical value of the grand canonical free energy, $\Omega_C[\phi]$, is given by its evaluation at the field configuration ϕ that satisfies the variational principle Eq. (4).

To obtain a variational functional for the particle densities, we artificially make the chemical potentials (fugacities) in the action Eq. (3) spatially dependent and thus transform the grand canonical free energy from a function to a functional

$$\Omega_C(\mu_j) \to \Omega_C[\mu_j(r)]. \tag{S.9}$$

This creates an additional degree of freedom in the choice in chemical potentials, which we

fix later by using the variational principle. This allows us to show that derivative,

$$
\begin{aligned}
-\frac{\partial \Omega_C}{\partial \mu_j(r)} &= -\frac{1}{\beta}\frac{1}{\mathcal{Z}}\frac{\partial \mathcal{Z}}{\partial \mu_j(r)} \\
&= \langle n_j(r) \rangle \equiv n_j(r),
\end{aligned}
\tag{S.10}
$$

is nothing but the functional average of the particle densities. If we look a the variational equation of the grand potential with respect to the particle density together with Eqs. (1) and (6), we find

$$
\begin{aligned}
\frac{\delta \Omega}{\delta n_j(r)} =& \frac{\delta \mathcal{F}_C}{\delta n_j(r)} - \tilde{\mu}_j \\
=& \frac{\partial \mu_j(r)}{\partial n_j(r)}\frac{\partial \Omega_C}{\partial \mu_j(r)} + \mu_j(r) + \frac{\partial \mu_j(r)}{\partial n_j(r)} n_j(r) - \tilde{\mu}_j \\
=& \mu_j(r) - \tilde{\mu}_j.
\end{aligned}
\tag{S.11}
$$

In order to find the true density distribution, we know that the chemical potentials must be constant and equal to the externally applied chemical potentials,

$$
\mu_j(r) = \tilde{\mu}_j, \tag{S.12}
$$

which means that the variational derivative above for the particle density is lifted to a variational principle,

$$
\frac{\delta \Omega_C}{\delta n_j(r)} = 0. \tag{S.13}
$$

In summary, the true particle density and potential distributions, according to equilibrium thermodynamics, are the ones with makes the functional $\Omega[n_j, \phi]$ stationary which is equivalent to Eq. (14).

2 Derivation of the chemical potentials

For the charge carriers, the particle density can be obtained as,

$$n_i(r) = -\frac{\partial \Omega_C}{\partial \mu_i(r)} = -\frac{\partial S[i^{-1}\phi]}{\partial \mu_i(r)} - \frac{1}{2\beta}\mathrm{tr} G \frac{\partial G^{-1}}{\partial \mu_i(r)}, \tag{S.14}$$

where the mean-field contribution is,

$$\frac{\partial S[i^{-1}\phi]}{\partial \mu_i(r)} = -e^{\beta \mu_i(r)} \Lambda_i^{-3} e^{-q_i \beta \phi(r)}, \tag{S.15}$$

and the one-loop contribution is,

$$\frac{\partial G^{-1}(r, r')}{\partial \mu_i(r)} = q_i^2 \beta^2 e^{\beta \mu_i(r')} \Lambda_i^{-3} e^{-q_i \beta \phi(r')} \delta(r - r') \delta(r - r'). \tag{S.16}$$

Together this gives the charge density,

$$n_i(r) = \Lambda_i^{-3} e^{\beta \mu_i(r)} e^{-q_i \beta \phi(r)} (1 - \beta \frac{q_i^2}{2} G(r, r)). \tag{S.17}$$

Inverted yields the chemical potential,

$$\mu_i(r) = \beta^{-1} \log \frac{n_i(r) \Lambda_i^3}{l_i(r)} + q_i \phi(r), \tag{S.18}$$

where we define the scaling parameter,

$$l_i(r) \equiv 1 - \beta \frac{q_i^2}{2} G(r, r). \tag{S.19}$$

A similar calculation for the solvent molecules yields,

$$\mu_s(r) = \beta^{-1}\log\left(\frac{n_s(r)\Lambda_s^3}{l_s(r)}\right) \tag{S.20}$$

$$- \beta^{-1}\log\left(\frac{\sinh(p\beta|\nabla\phi(r)|)}{p\beta|\nabla\phi(r)|}\right), \tag{S.21}$$

with,

$$l_s(r) \equiv 1 + \frac{\beta p^2}{2}(\mathcal{L}^2 + \mathcal{L}')\nabla^2 G(r,r). \tag{S.22}$$

3 Derivation of the second variational derivative

Starting from the action, Eq. (3), the first variational derivative is exactly the Euler-Lagrange equation, which reads,

$$\frac{\delta S[\psi]}{\delta\psi(r')} = i\rho_{\text{ext}}(r') + i\sum_{i=a/c} q_i\lambda_i\Lambda_i^{-3}e^{-iq_i\beta\psi(r')} - \nabla\Big(\epsilon(r')\nabla\psi(r')\Big), \tag{S.23}$$

with

$$\epsilon(r') = \epsilon_0 - \frac{ip\beta\lambda_s\Lambda_s^{-3}}{|\nabla\psi(r')|}\frac{\sinh(ip\beta|\nabla\psi(r')|)}{ip\beta|\nabla\psi(r')|}\mathcal{L}(ip\beta|\nabla\psi(r')|). \tag{S.24}$$

The second variational derivative can be computed in a similar fashion, using a generalized Euler-Lagrange equation or simply by using the rules of functional differentiation which

yields

$$\frac{\delta^2 S[\psi]}{\delta\psi(r)\delta\psi(r')} = \beta \sum_{i=a/c} q_i^2 \lambda_i \Lambda_i^{-3} e^{-iq_i\beta\psi(r')}\delta(r-r') - \nabla\left(\frac{\delta\epsilon(r')}{\delta\psi(r)}\nabla\psi(r') + \epsilon(r')\nabla\delta(r-r')\right)$$

$$= \beta \sum_{i=a/c} q_i^2 \lambda_i \Lambda_i^{-3} e^{-iq_i\beta\psi(r')}\delta(r-r')$$

$$- \nabla\left(\left(\epsilon_0 + \lambda_s\Lambda_s^{-3}\beta^2 p^2 \frac{\sinh ip\beta|\nabla\psi(r')|}{ip\beta|\nabla\psi(r')|}\left(\mathcal{L}^2 + \mathcal{L}'\right)\right)\nabla\delta(r-r')\right). \tag{S.25}$$

If we now evaluate the second variational derivative at the electric potential Eq. (S.1), like it is stated in the result for the grand canonical free energy Eq. (4), and insert our results for the chemical potentials Eqs. (8) and (9), we obtain the differential equation for the correlation function Eq. (12).

4 Details on the correlation parameters

The solution of the differential equation Eq. (12), for spatially independent fields, follows closely the works of Levy et al. who studied Coulombic correlations in bulk electrolytes.[S2]

The equal point Green's function and its Laplacian can be obtained by, assuming spatially constant fields, and Fourier transforming Eq. (12), which allows us to compute,

$$G(r,r) = \frac{1}{\epsilon(r)} \int_{|k|<k_{max}} \frac{d^3k}{(2\pi)^3} \cdot \frac{1}{\boldsymbol{k}^2 + \lambda_D^{-2}} \tag{S.26}$$

$$= \frac{1}{2\pi^2\epsilon(r)}\left(\frac{2\pi}{\Lambda_B} - \frac{1}{\lambda_D}\arctan\left(2\pi\frac{\lambda_D}{\Lambda_B}\right)\right), \tag{S.27}$$

and

$$\nabla^2 G(r,r) = -\int_{|k|<k_{max}} \frac{d^3k}{(2\pi)^3}\boldsymbol{k}^2 \tilde{G}(k) \tag{S.28}$$

$$= -\frac{1}{2\pi^2\epsilon(r)}\left(\frac{8\pi^3}{3\Lambda_B^3} - \frac{2\pi}{\lambda_D^2\Lambda_B} + \frac{1}{\lambda_D^3}\arctan\left(2\pi\frac{\lambda_D}{\Lambda_B}\right)\right). \tag{S.29}$$

where a maximum wavelength cutoff $k_{max} = 2\pi/\Lambda_B$ was introduced to fix the divergence of the Green's function at equal argument. This large-momentum cutoff corresponds to a small-distance cutoff.

The solution for the equal point correlation functions inserted into Eqs. (10) and (11), we obtain the correlation parameters

$$\begin{aligned} l_i(r) &= 1 - \frac{\beta q_i^2}{4\pi^2\epsilon(r)} \\ &\left(\frac{2\pi}{\Lambda_B} - \frac{1}{\lambda_D}\arctan\left(2\pi\frac{\lambda_D}{\Lambda_B}\right)\right), \end{aligned} \tag{S.30}$$

$$\begin{aligned} l_s(r) &= 1 - \frac{\beta p^2}{4\pi^2\epsilon(r)}(\mathcal{L}^2 + \mathcal{L}') \\ &\left(\frac{8\pi^3}{3\Lambda_B^3} - \frac{2\pi}{\lambda_D^2\Lambda_B} + \frac{1}{\lambda_D^3}\arctan\left(2\pi\frac{\lambda_D}{\Lambda_B}\right)\right). \end{aligned} \tag{S.31}$$

where $\epsilon(r)$ is a prefactor in Eq. (12) and $\lambda_D = \sqrt{\epsilon(r)/(\beta\sum_{i=a/c} q_i^2 n_i(r))}$ is a spatially dependent Debye length and Λ_B is the small distance cutoff.

5 Details of free energy functionals

The metal free energy functional is decomposed in three terms,

$$\mathcal{F}_Q = \int_r T_e + U_{ex} + U_c, \tag{S.32}$$

with T_e being the kinetic energy from Thomas-Fermi theory,[S3] which depends on the electron density and its gradients with

$$T_e = e_{au} a_0^{-3} t_{TF}(1 + \theta_T s^2). \tag{S.33}$$

U_{ex} as well as U_c are exchange and correlation terms from the Perdew-Burke-Ernzerhof functional, which is a generalized gradient approximation,[S3,S4] which have the form,

$$U_{ex} = e_{au} a_0^{-3} u_{ex} (1 + \theta_{ex} s^2), \tag{S.34}$$

$$U_c = e_{au} a_0^{-3} (u_c + \theta_c n_e a_0^3 t^2). \tag{S.35}$$

Here $t_{TF} \sim n_e^{4/3}$ is the volumetric kinetic energy, $u_{ex} \sim n_e^{4/3}$ is the volumetric exchange energy and u_c is an interpolation of the volumetric correlation energy of an uniform electron gas. The factors $s \sim |\nabla n_e|/n_e^{4/3}$ and $t \sim |\nabla n_e|/n_e^{7/6}$ are corrections for the gradient terms in the kinetic- and exchange correlation energies. Thus the parameters θ_T, θ_{ex} and θ_c are tuning the strengths of the gradient coefficients. These parameters need to be determined through simulation or experimental data, e.g. for an AG(111)-KPF_6 aqueous interface.[S5] Here, $e_{au} = e_0^2/(4\pi\epsilon_0 a_o)$ is the atomic energy and a_0 the Bohr radius.

The interaction free energy is,

$$\mathcal{F}_{\text{int}} = \int_r \sum_{j=a/c/s} n_j W_j, \tag{S.36}$$

where W_j is a parametrizable Lennard Jones potentials of the form,

$$W_j(r) = \omega_j \cdot \left(\frac{\sigma_j^1}{d\text{M}(r) \cdot \Theta(d\text{M}(r)) + \sigma_j^2} \right)^6, \tag{S.37}$$

where ω_j is a positive number describing the repulsion force. The two length scales σ_j^1 and σ_j^2 describe range of interaction. The function $d\text{M}(r)$ is the closest distance to the metal at position r, which is negative when r is in the metal and positive if it is outside of the metal. Excess free energies describe non electrostatic interactions such as hard-core repulsion and vary in complexity. In this model we account for steric effects using the excess free energy of an ideal lattice gas, where all particles occupy a lattice with density n_{max}.[S6,S7] For the steric

free energy on the level of the Bikerman theory one has a chemical potential of

$$\frac{\partial \mathcal{F}_{\mathrm{st}}}{\partial n_j} = \beta^{-1} \log \left(\frac{1}{1 - \sum_{j'} n_{j'}/n_{\mathrm{max}}} \right). \tag{S.38}$$

6 Model parameters

Corresponding to the Ag(111) electrode, the metal parameters are taken from Huang et al.[S8] The metal-electrolyte interaction described by the Lennard Jones Potential, in Eq. (S.37), is parameterized by a single length scale which determines the closest distance to the metal surface and is taken to be 4 $a_0 \approx 2$ Å, where a_0 is the Bohr radius. The cutoff is physically a lengthscale, above which Coulombic correlation effects are accounted for. It is bounded from below by the pariticle's size and from above by the Bjerrum length.[S9–S11] In our model, the modified Bjerrum length,

$$\lambda_B(r) = \frac{\beta e^2}{4\pi\epsilon(r)}, \tag{S.39}$$

is spatially dependent due to the varying prefactor, $\epsilon(r)$, in the differential equation for the correlation function, Eq. (12). We found that it ranges from 0.7 nm in the solution bulk to $\mathcal{O}(10)$ nm at the interface. In principle we could have chosen the cutoff to be equal to the modified Bjerrum length, thus spatially dependent, but for simplicity we chose a constant value of $\Lambda_B \equiv \lambda_B = 3.5$ nm. Studying the dependence of our results on the cutoff parameter showed that the qualitative behaviour (shape) of corrections was not affected, however, the magnitude of corrections was increasing, with decreasing cutoff, as expected. For infinite cutoff, the one-loop solution reduces to the mean-field model. In this sense, the cutoff length can be seen as a fitting parameter that determines the strength of correlation effects in the model system.

Water molecules are considered with a radius of 1.35 Å and effective dipole moment of 4.7 D. Anions and cations, including a solvation shell, have a radius of 3 Å and 4 Å

respectively. The chosen value of the effective water dipole moment ensures the correct bulk water permittivity in the point-dipole model. We have an ionic bulk concentration of 100 mM and a solvent concentration of 55.6 M, which, together with the effective dipole moment, gives a water bulk permittivity, Eq.(17) for zero electric field, of around 80. The external

Table S1: Model parameters for the electrolyte.

Symbol	Description	Value
Solution		
n_{ion}^{Bulk}	Ionic bulk concentration	100 mM
n_{s}^{Bulk}	Solvent bulk concentration	55.6 M
r_a	Anion radius	3 Å
r_c	Cation radius	4 Å
r_s	Solvent radius	1.35 Å
z_a	Anion charge	-1
z_c	Cation charge	$+1$
p	Effective water dipole moment	4.7 D
Λ_B	Cutoff	3.5 nm
Metal-Solution-Interaction		
ω_j	Short-range force constant for particle j	10 $\mathrm{k_B T}$
σ_j^1	Closest distance for electrolyte	4 a_0
σ_j^2	Steepness of Lennard Jones Potential j	$\sigma_j^1/5$

parameters fixed in the grand-canonical ensemble, are on the one hand the electrochemical potentials of the solution species $\tilde{\mu}_j$, determined by the species bulk density and on the other hand the electrochemical potential of the electrons $\tilde{\mu}_e$ which is equivalent to the electrode potential. This can be seen from Eq. (1).

References

(S1) Netz, R.; Orland, H. Beyond Poisson-Boltzmann: Fluctuation Effects and Correlation Functions. *Eur. Phys. J. E* **2000**, *1*, 203–214.

(S2) Levy, A.; Andelman, D.; Orland, H. Dielectric Constant of Ionic Solutions: A Field-Theory Approach. *Phys. Rev. Lett.* **2012**, *108*, 227801.

(S3) Thomas, L. H. The Calculation of Atomic Fields. *Math. Proc. Camb. Philos. Soc.* **1927**, *23*, 542–548.

(S4) Perdew, J. P.; Burke, K.; Ernzerhof, M. Generalized Gradient Approximation Made Simple. *Phys. Rev. Lett.* **1996**, *77*, 3865–3868.

(S5) Huang, J. Density-Potential Functional Theory of Electrochemical Double Layers: Calibration on the Ag(111)-KPF6 System and Parametric Analysis. *J. Chem. Theory Comput.* **2023**,

(S6) Bikerman, J. J. Structure and Capacity of Electrical Double Layer. *Philosophical Magazine* **1942**, *33*, 384–397.

(S7) Kornyshev, A. A. Double-Layer in Ionic Liquids: Paradigm Change? *J. Phys. Chem. B* **2007**, *111*, 5545–5557.

(S8) Huang, J.; Chen, S.; Eikerling, M. Grand-Canonical Model of Electrochemical Double Layers from a Hybrid Density Potential Functional. *J. Chem. Theory Comput.* **2021**, *17*, 2417–2430.

(S9) Santangelo, C. D. Computing Counterion Densities at Intermediate Coupling. *Phys. Rev. E* **2006**, *73*, 041512.

(S10) Bazant, M. Z.; Storey, B. D.; Kornyshev, A. A. Double Layer in Ionic Liquids: Overscreening versus Crowding. *Phys. Rev. Lett.* **2011**, *106*, 046102.

(S11) Storey, B. D.; Bazant, M. Z. Effects of Electrostatic Correlations on Electrokinetic Phenomena. *Phys. Rev. E* **2012**, *86*, 056303.
